# Collaborative intelligence and gamification for on-line malaria species differentiation

**DOI:** 10.1186/s12936-019-2662-9

**Published:** 2019-01-24

**Authors:** María Linares, María Postigo, Daniel Cuadrado, Alejandra Ortiz-Ruiz, Sara Gil-Casanova, Alexander Vladimirov, Jaime García-Villena, José María Nuñez-Escobedo, Joaquín Martínez-López, José Miguel Rubio, María Jesús Ledesma-Carbayo, Andrés Santos, Quique Bassat, Miguel Luengo-Oroz

**Affiliations:** 1 Research Institute Hospital 12 de Octubre/CNIO, Universidad Complutense de Madrid, Ciudad Universitaria, 28040 Madrid, Spain; 2 Biomedical Image Technologies Group, DIE, ETSI Telecomunicación, Universidad Politécnica de Madrid, CEI Moncloa UPM-UCM, Madrid, Spain; 3 Malaria and Emerging Parasitic Diseases Laboratory, National Microbiology Centre, Instituto de Salud Carlos III, Madrid, Spain; 4 Centro de Investigación Biomédica en Red en Bioingeniería, Biomateriales y Nanomedicina (CIBER-BBN), Madrid, Spain; 5 ISGlobal, Hospital Clínic, Universitat de Barcelona, Barcelona, Spain; 6 Centro de Investigação em Saúde de Manhiça (CISM), Maputo, Mozambique; 7 ICREA, Pg. Lluís Companys 23, 08010 Barcelona, Spain; 8 Pediatric Infectious Diseases Unit, Pediatrics Department, Hospital Sant Joan de Déu, University of Barcelona, Barcelona, Spain

**Keywords:** Crowdsourcing, Malaria classification, Image analysis, Games for health, Telepathology

## Abstract

**Background:**

Current World Health Organization recommendations for the management of malaria include the need for a parasitological confirmation prior to triggering appropriate treatment. The use of rapid diagnostic tests (RDTs) for malaria has contributed to a better infection recognition and a more targeted treatment. Nevertheless, low-density infections and parasites that fail to produce HRP2 can cause false-negative RDT results. Microscopy has traditionally been the methodology most commonly used to quantify malaria and characterize the infecting species, but the wider use of this technique remains challenging, as it requires trained personnel and processing capacity.

**Objective:**

In this study, the feasibility of an on-line system for remote malaria species identification and differentiation has been investigated by crowdsourcing the analysis of digitalized infected thin blood smears by non-expert observers using a mobile app.

**Methods:**

An on-line videogame in which players learned how to differentiate the young trophozoite stage of the five *Plasmodium* species has been designed. Images were digitalized with a smartphone camera adapted to the ocular of a conventional light microscope. Images from infected red blood cells were cropped and puzzled into an on-line game. During the game, players had to decide the malaria species (*Plasmodium falciparum*, *Plasmodium malariae, Plasmodium vivax*, *Plasmodium ovale*, *Plasmodium knowlesi*) of the infected cells that were shown in the screen. After 2 months, each player’s decisions were analysed individually and collectively.

**Results:**

On-line volunteers playing the game made more than 500,000 assessments for species differentiation. Statistically, when the choice of several players was combined (n > 25), they were able to significantly discriminate *Plasmodium* species, reaching a level of accuracy of 99% for all species combinations, except for *P. knowlesi* (80%). Non-expert decisions on which *Plasmodium* species was shown in the screen were made in less than 3 s.

**Conclusion:**

These findings show that it is possible to train malaria-naïve non-experts to identify and differentiate malaria species in digitalized thin blood samples. Although the accuracy of a single player is not perfect, the combination of the responses of multiple casual gamers can achieve an accuracy that is within the range of the diagnostic accuracy made by a trained microscopist.

**Electronic supplementary material:**

The online version of this article (10.1186/s12936-019-2662-9) contains supplementary material, which is available to authorized users.

## Background

In 2016, an estimated 216 million cases of malaria occurred worldwide, leading to 445,000 deaths. *Plasmodium falciparum* remains the most prevalent malaria parasite in sub-Saharan Africa, accounting for 99% of estimated malaria cases. Outside of Africa, *P. vivax* is the predominant parasite in America, representing 64% of malaria cases, accounting for over 30% and 40% of the cases in the South East Asia and Eastern Mediterranean regions, respectively [1]. *Plasmodium malariae* is widely distributed and it can be responsible for a significant proportion of malaria cases in some American regions [2]. The *Plasmodium ovale* distribution is highest in Western Africa, potentially accounting for up to 10% of the cases [3, 4]. Finally, *Plasmodium knowlesi* is cause of human malaria in Malaysia and is increasing in other Southeast Asian countries. Due its similarities with *P. falciparum*, misidentification is common [5, 6].

Prompt diagnosis and treatment of malaria patients is the most effective intervention to avoid severe disease and reduce malaria transmission. World Health Organization recommends that every suspected malaria case be confirmed by microscopy or a rapid diagnostic test (RDT) before treatment [1, 3]. Nevertheless, low densities and parasites that fail to produce histidine-rich protein 2 (HRP2), one of the common antigens detected by RDT, can cause false-negative RDT results [1, 7]. Giemsa-stained thick and thin films of blood examined by light microscopy not only enables to confirm infection and estimate the parasitaemia levels, but also, identify the *Plasmodium* species [5, 8, 9].

The correct classification of the species at the time of diagnosis is important for adequately tailoring the malaria treatment, as different species may require different therapeutic approaches [3, 5, 8, 10, 11]. Most commonly, artemisinin-based combination treatment (ACT) is used for *P. falciparum* and *P. vivax* in regions where chloroquine resistance is prevalent whereas chloroquine is still used in most settings for non-falciparum species. Moreover, *Plasmodium vivax* and *P. ovale* require the addition of an anti-hypnozoite treatment (primaquine or tafenoquine) to prevent relapses [5, 12–14].

Previous reports have highlighted the challenges of correctly identifying the different malaria species [3, 4, 15, 16]. In a context where the relative contribution of the non-falciparum *Plasmodium* species to the global malaria burden seems to be growing, and where severe malaria cases are increasingly associated to *P. vivax* or even *P. knowlesi* [3, 5, 15, 17–19], it is important to improve their differentiation. Malaria classification by light microscopy requires trained microscopy technicians, capable of detecting and identifying parasites with precision, using parasite and red blood cells morphological characteristics such as size, shape and pigmentation, a process that is time consuming, labor intensive and moderately complex. This time and expertise required could delay the correct diagnosis of hundreds of people during the high season [5, 20–22].

Crowdsourcing methodologies using the power of citizen connected via the Internet have been recently developed to solve scientific challenges involving “big data” analyses that cannot be entirely automated, improving the quality, cost, and speed of certain complex procedures. These contributions can be achieved with different motivation strategies, such as the gamification approach which intends, not only to entertain users, but also to train or educate them. Systems based on crowdsourcing strategies have been applied to solve public problems, including health challenges providing a promising complement to traditional methods [21, 23–27]. For instance, 165,000 citizen scientists have used the EyeWire platform to map the three-dimensional structure of retinal neurons. This mapping provides the first glimpse of how the structure and organization of neurons function to detect motion [28].

In 2012 two research groups independently developed two on-line crowdsourcing platforms Biogames [22] and MalariaSpot [29, 30] to diagnose malaria infected red blood cells using Giemsa-stained blood smears imaged under light microscopes. ‘‘BioGames’’ is a web-based training environment that teaches players to identify malaria-infected red blood cells in digitalized thin blood smears. The evaluation of an untrained crowd of gamers showed a similar diagnostic accuracy of trained diagnosticians [22]. The MalariaSpot game [29] showed that the combination of the parasite counts made by 20 on-line volunteers playing over the digitalized thick blood smear could achieve a parasite counting accuracy that is within that of the diagnostics decisions made by a trained microscopist too.

In this study, the previous approach has been adapted and a system for remote malaria image analysis has been developed in order to discover if non-experts can make a correct identification of five different parasite species from digitalized thin blood smears. Moreover, this study proposes a methodology to combine multiple opinions from different on-line observers into one single collaborative decision and the time that is taken to reach these decisions has been measured.

## Methods

A videogame (“MalariaSpot Bubbles”) with embedded puzzles in which the gamers have to learn how to classify the five species of parasites (*Plasmodium* species: *P. falciparum*, *P. malariae, P. vivax*, *P. ovale*, *P. knowlesi*) has been developed using cropped images digitalized from thin blood films. The game had 4 levels of increasing difficulty, with players having to differentiate between two species on the first level, and up to five species in the last difficulty level (level 4). All the players’ decisions were registered in a database. After 2 months, all the collected data were processed in order to analyse the accuracy of the players to differentiate among species.

### Digitalization of clinical samples

The malaria slides used in this study were obtained from the Malaria and Emerging Parasitic Diseases Laboratory (Spanish National Biobanks Registry No: C.0001392). All slides were previously re-examined and verified by expert in the field, as well as confirmed by polymerase chain reaction (PCR) to give a species-specific diagnosis.

Twenty Giemsa-stained anonymous thin blood smears were digitalized using a mobile phone (Sony Xperia Z2) coupled to an adaptor (Universal Digiscoping, Cellscope) as previously described by Ortiz et al. [15]. Images were acquired with the “Camera FV-5 Pro” mobile application, under the oil immersion objective of the microscope Zeiss AX05COP2) in PNG format with resolution of 3.1 Mpx. Camera FV-5 Pro is a camera application for android mobile devices that allowed to define a standardized acquisition protocol by selecting stable parameters for exposure compensation, ISO, light metering mode, focus and white balance based on the qualitative assessment of ten experts on malaria microscopy. A total of 114 images of young trophozoites which represented with more confidence the species-specific parameters were chosen from this image repository, excluding those samples with presence of mixed infections, bad staining or those highly deteriorated. To build the set of images used in the game, each individual parasite was cropped to fixed dimensions of 190 × 190 pixels.

### Game architecture

**Fig. 1:**
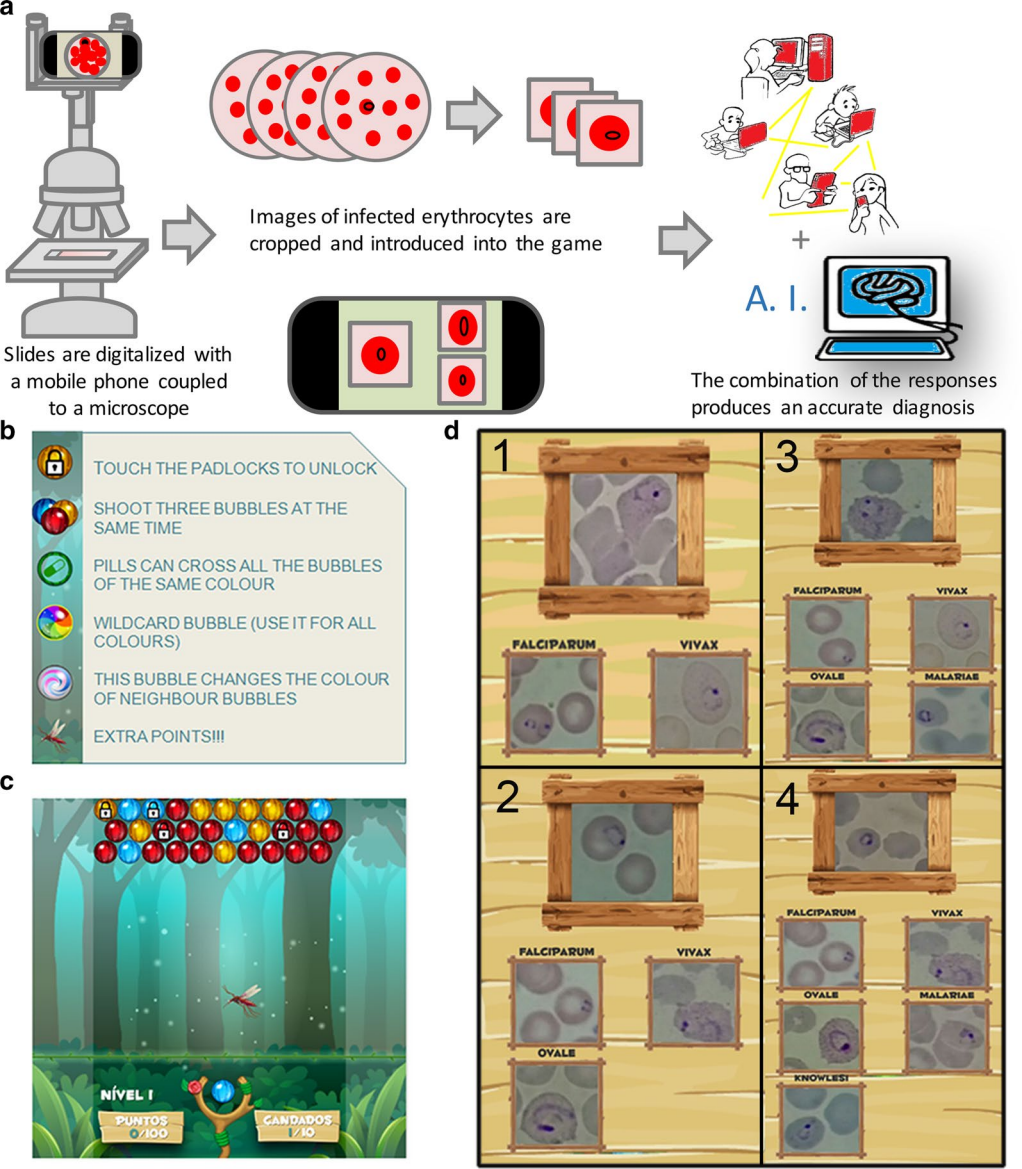
**a** Representative scheme of the process. **b** Instructions briefly explained in the tutorial shown at the beginning of the game. **c** Screen with unlocked bubbles to shoot and bubbles with padlocks to unlock. **d** Example of puzzle in level 1, 2, 3 and 4, respectively

The objective of MalariaSpot Bubbles was to “shoot” colour bubbles and solve puzzles that required identifying the malaria species of an infected red blood cell to score points and pass levels. The game could be played on-line or be downloaded as an app for iOS or Android smartphones. To make the game more entertaining, the user was presented with a quest: to save mankind from malaria hunting the five malaria species in different countries of the world. A representative scheme of the process is seen in Fig. 1a.

The instructions were briefly explained in the tutorial option at the beginning (Fig. 1b). During the game (Fig. 1c) players were confronted to puzzles that consisted of a screen in which the image of the parasite to diagnose was shown. Then, gamers had to choose the right parasite species using prototypical reference images (chosen randomly each time) as support (Fig. 1d). Once gamers had solved enough puzzles and scored enough points, they could progress to the next level. In the first level, they had to discriminate between *P. falciparum* and *P. vivax* and solve 10 puzzles. At level two, *P. ovale* was introduced and gamers had to learn to differentiate among the three species. In this level they had to solve 15 puzzles. For the third level the new species was *P. malariae* and there was a minimum of 20 puzzles to solve. In the fourth and last level gamers were introduced to *P. knowlesi* and 25 puzzles had to be solved to differentiate among the 5 species. Before starting each level, the users were presented with a screen showing the *Plasmodium* species presented at this level, highlighting differences in shape and size (Additional file 1: Figure S1a–e). Players were given continuous feedback: after every success or mistake they were shown the right solution (Additional file 1: Figure S1f–g). At the end of the game, scores were shown (Additional file 1: Figure S1h) and players were invited to register in the high scores table.

### Data collection and preprocessing

All the player´s decisions from each puzzle or species classification were transmitted in real time by the videogame to the cloud and stored in a custom-made database using the backend as a service Parse and the open-standard JSON format. Data contained the image identification number, puzzle identification number, if the response was true or false, response chosen by the gamer, correct solution, level, time to response, platform (android, ios, webgl) and nickname of the player.

### Statistical analysis

All statistical analyses were performed using SPSS (version 23) and the GraphPad Prism Software (version 6). Contingency tables were used to analyse associations between categorical variables, considering Fisher exact test or Chi square test for statistical significance. The parametric T-Student test or the non-parametric test U-Mann–Whitney were used to compare averages of continuous variables between two groups and the parametric analysis of variance (ANOVA) Test or the non-parametric test Kruskal–Wallis were used when more of two groups were compared. Chi Square test was used for categorical variables. P values ≤ 0.05 were considered statistically significant.

### Collaborative species classification

A simulation environment using Matlab was created in order to assess the performance of a potential real-time system that could use the analysis made by multiple players over the same image. In particular, for each image sample and for different sizes of the group of players analysing the same image, the decisions made by players were recreated and provided a collective decision based on the parasite species which was selected more times by the players conforming the group. The simulation environment allowed to replicate the experiment multiple times with different groups for each sample [29].

### Ethics

No ethical review board approval was required since the images used in our work were not linked to any patient data or diagnosis and the samples belong to the Spanish National Biobanks (Registry No: C.0001392). Data analysed in this research were anonymously produced by on-line volunteers who agreed to play an on-line internet game. Participants were informed of the research purposes of the game.

## Results

### Videogame and crowdsourcing architecture

A videogame and a backend architecture with servers and databases on the cloud that allowed to run the experiments in real time was developed, showing new image samples to the players and collecting all the information that they produced in real time. After multiple in-house tests, MalariaSpot Bubbles was made available to the public on April 25, 2016 (World Malaria Day). In July 2016, more than 25,000 people from 121 different countries around the world had played MalariaSpot Bubbles. In this period, gamers played a total of 596,235 puzzles over the 114 images samples tested in the game and generating a database of more than half a million species classification decisions.

### Players’ behaviour

**Fig. 2:**
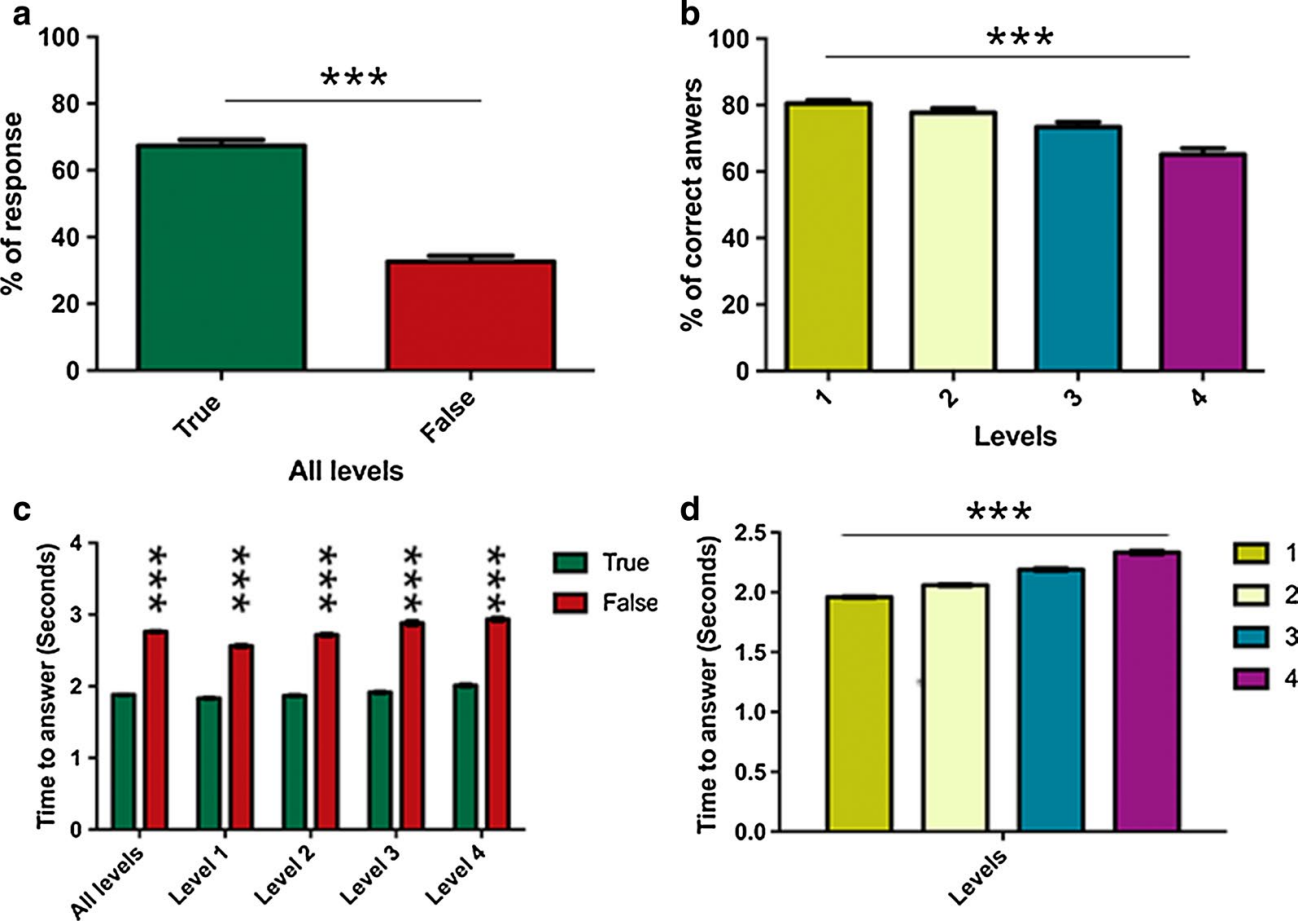
**a** Percentage of correct (true, green) or incorrect (false, red) answers of players when all the levels were analysed together. **b** Percentage of correct answers of gamers in the different levels of difficulty. **c** Time to answer when gamers hit the response (true, green) or not (false, red) in global (all levels) and in the 4 different levels of difficulty. **d** Time to answer in the four levels of difficulty. Values given represents the mean ± SEM. ***P ≤ 0.0001

Out of a total of 596,235 decisions, 447,176 (75%) tagged the correct species. Gamers were able to identify the species correctly most of the times in all the levels, but performance decreased with the number of options (level) as expected (Fig. 2a, b). The digital nature of the classification task allowed to quantify the time from the moment that the image of the infected blood sample is shown to the moment when the decision is taken. The mean time to decide the malaria species shown in the image was 2.09 s and depended on the level of difficulty itself, ranging from 1.96 s for 2 species to 2.33 s if the decision was between 5 possible species (Fig. 2d). Interestingly, gamers spent less time when they choose the correct species in all the levels analysed (Fig. 2c). The percentage of correct classifications increased as players performed more classifications (Additional file 2: Figure S2a) and they spent less time (Additional file 2: Figure S2b). The percentage of success also increased when players have beaten the four levels of difficulty (Additional file 2: Figure S2c).

### Species differentiation

**Fig. 3:**
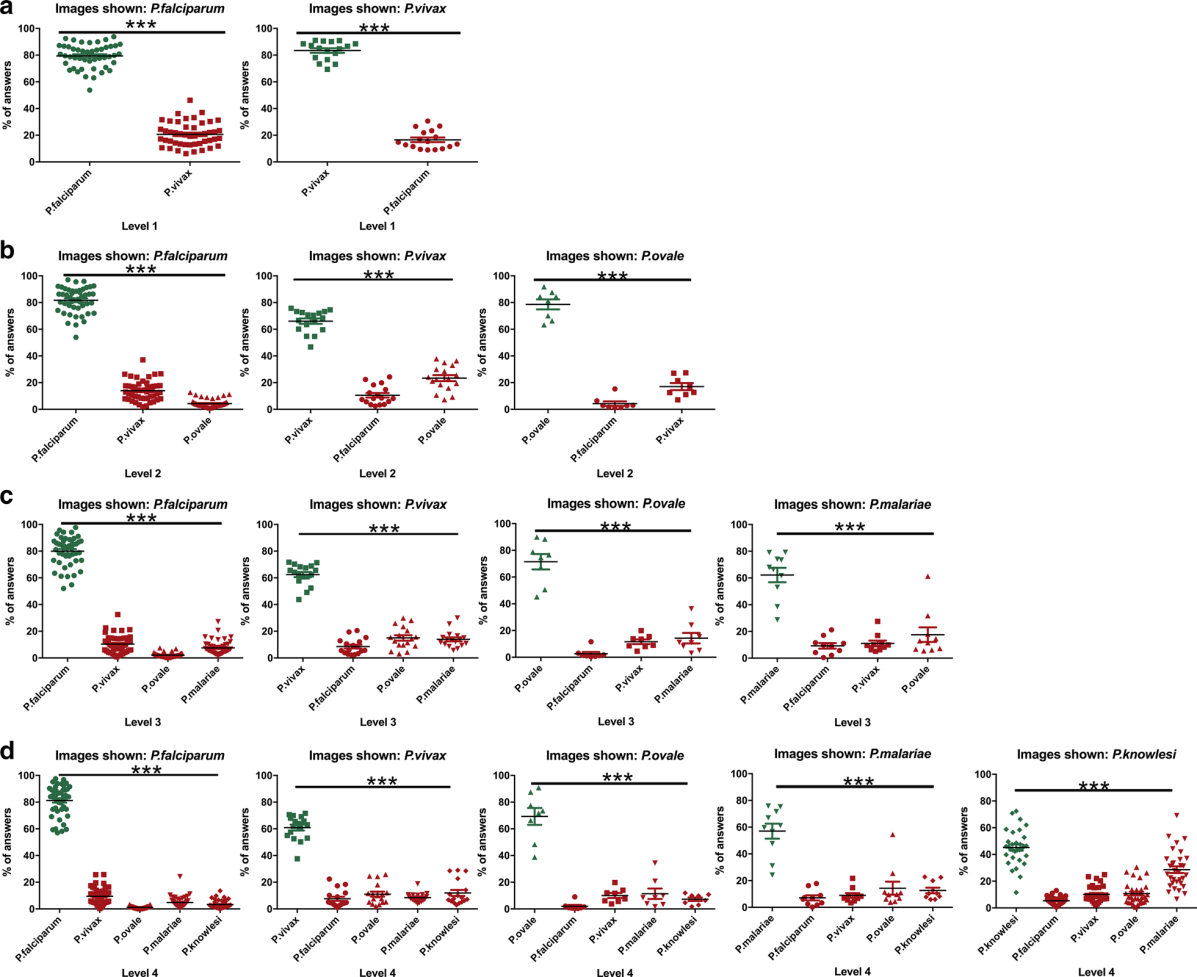
Percentage of answers for each species shown at level 1 (**a**), 2 (**b**), 3 (**c**) and 4 (**d**). The correct solution is represented in green and false answers in red. Values given represents the mean ± SEM. ***P ≤ 0.0001

Gamers accurately differentiated the five malaria species (P ≤ 0.0001). It is important to note that the probability of success was higher at level 1 (when only *P. falciparum* and *P. vivax* were shown) than at level 4 (when all the species were shown at the same time). Results were analysed independently for every level (Fig. 3).

At level 1, 116,080 decisions were obtained for questions related to *P. falciparum* and 116,080 answers for *P. vivax*. Both species were significantly differentiated by gamers (P ≤ 0.0001). The percentages of correct answers were 79% and 83% for *P. falciparum* and *P. vivax*, respectively. The specificity of this level was 99.9% for both species.

At level 2, the percentage of success for the new introduced species, *P. ovale,* reached 79%. Nevertheless, the number of hits for *P. vivax* decreased to 66% (which was mistaken with *P. ovale* in 23% of the cases). The successful identification of *P. falciparum* was similar to level 1 (82% of correct answers). In this level, 163,661 decisions were registered: 54,358 for *P. falciparum* images, 54,542 for *P. vivax* and 54,761 for *P. ovale*. Again, the three species were correctly differentiated by gamers (P ≤ 0.0001) and a specificity of diagnosis of 99.9% was obtained.

At level 3, users were still capable of significantly distinguishing the four species shown (P ≤ 0.0001). A specificity of diagnosis of 99.9% for *P. falciparum, P. vivax* and *P. ovale* and of 90% for *P. malariae* was achieved. The level of success for the three species which had been already introduced was similar to the observed at level 2 (80% of hits for *P. falciparum* with a total of 29,185 clicks, 71% for *P. vivax* with a total of 29,392 decisions and 62% for *P. ovale* with a total of 29,596 clicks). For the new species, *P. malariae*, a total of 29,920 answers were registered, with a 62% success rate.

At the last and most difficult level, gamers continued differentiating the five species shown (P ≤ 0.0001). The same level of hits was obtained for *P. falciparum* (81% for a total of 16,344 decisions); *P. ovale* (69% for a total of 16,689); *P. vivax* (61% for a total of 16,612) and *P. malariae* (57% for a total of 16,562). The most challenging species to differentiate at level 4 was *P. knowlesi,* with a 45% of hits over 16,490 decisions. This newly introduced species was mostly mistaken with *P. malariae* (in a 29% of the cases), although both species were significantly differentiated (P ≤ 0.0001). The level of specificity of this level was 99.9% for *P. falciparum*, *P. vivax* and *P. ovale*, 90% for *P. malariae* and 81% for *P. knowlesi*.

### Collaborative species classification

**Fig. 4:**
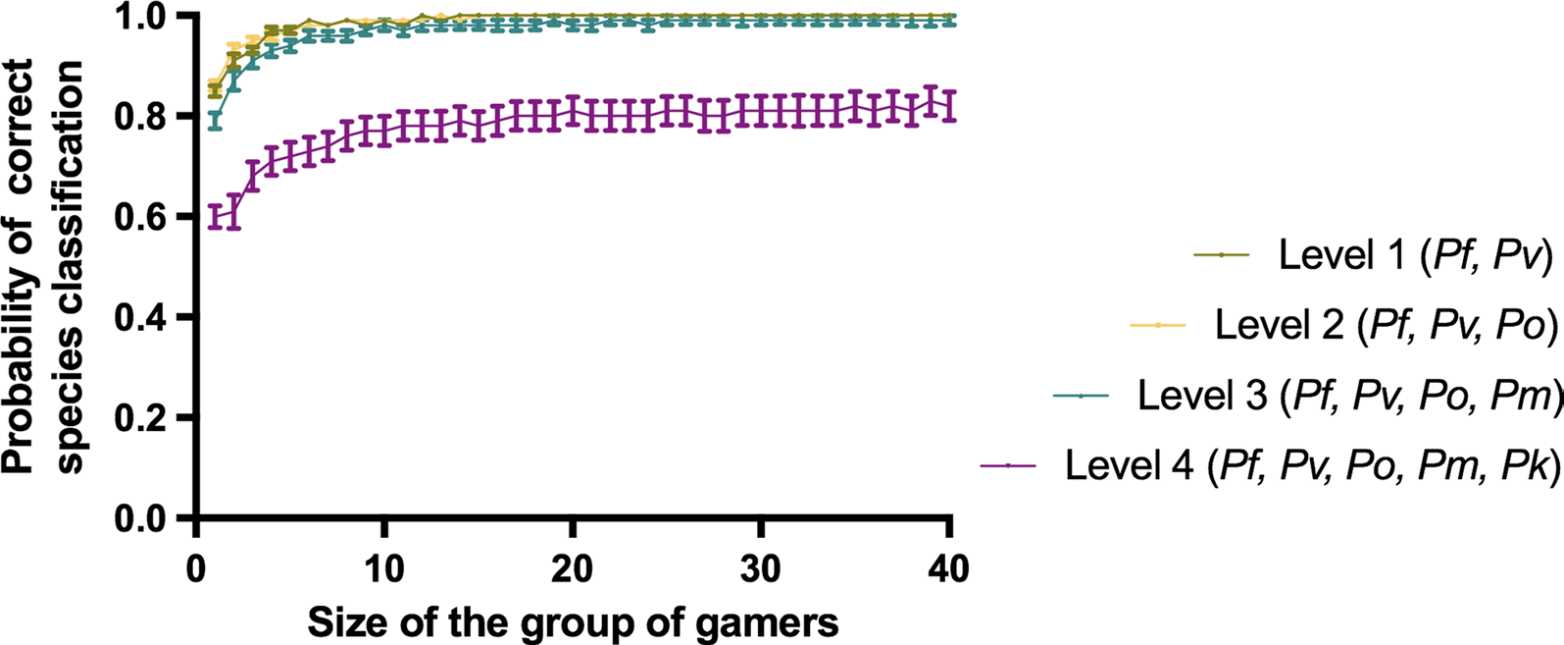
Model of the collective identification of malaria species. Curve fitting of the mean accuracy rate for all group sizes (1–40) and training levels. Each point represents the average ± SEM from 30 measures

Finally, the minimum number of on-line analysts over the same sample that would be needed to obtain an accurate diagnosis in a hypothetical real time system was evaluated. For this, 30 simulations of the analysis of each of the images samples for group sizes from 2 to 40 gamers in each of the levels were performed. In each simulation, the individual decisions of a random group of players of a certain size are combined into a collective decision by a voting algorithm that chooses the species which obtains more votes (Fig. 4). For level 1 (*P. falciparum* and *P. vivax*), the probability of correct species classification reached 99.9% when the answers of a minimum of 14 gamers were combined. For level 2 (*P. falciparum, P. vivax* and *P. ovale*), it reached 99.9% when the group of gamers had a minimum size of 15. When 4 species where compared (level 3: *P. falciparum, P. vivax, P. ovale* and *P. malariae*), the maximum probability reached was 99%. This probability was obtained when the size of the group was composed of at least of 25 gamers. For the most difficult level (level 4: *P. falciparum, P. vivax, P. ovale, P. malariae* and *P. knowlesi*), the maximum probability reached was 80%, obtained when combining the answers of at least 17 gamers.

## Discussion

This study reports results of an innovative on-line game platform for classifying malaria parasites from images of thin blood smears digitalized by a smartphone coupled to a conventional light microscope. Results extracted from more than half a million malaria species classifications attempts using an on-line app made by on-line volunteers showed that combining the answers from different players, the diagnosis obtained can be as accurate as the one obtained by an expert, especially for *P. falciparum*, *P. vivax* and *P. ovale*. Volunteers experienced more difficulties when they had to distinguish between *P. malariae* and *P. knowlesi* classification, a finding also experienced among expert microscopists in real life, as trophozoites of both species are very similar under the microscope. Previous works report the difficulty for differentiating these latter two species from one to another and from the others [12, 15, 31–33]. An earlier introduction of the species or a different training strategy could facilitate the learning process, as it has been reported in areas non-familiarized with the non-falciparum infections [3]. In this line, the applications of such system could be also used for training and educational purposes for students and field workers to improve the diagnosis performance. It is also worth noting the strong impact of the projects in terms of sensitization since in only a few months, more than 25,000 people were exposed to information regarding malaria and its diagnosis. In this study, in order to simplify the complexity of the game, only the early trophozoite forms of the five malaria species have been evaluated. Nevertheless, as demonstrated in a previous work, non-expert volunteers are able to differentiate the four parasite stages, although they presented more difficulties to discriminate gametocytes [15]. Further studies are necessary to include late stage trophozoites, schizonts and gametocytes.

In addition to capacity building and advocacy applications, the proposed approach could offer an alternative or complementary approach to classify malaria species using remote digital analysis. This system could use a mobile phone coupled to a regular microscope or directly a mobile microscope to digitalize the sample and transmit it to the internet over the mobile network which distributes the images to enough on-line examiners and produces an aggregate parasite classification and quantification that is sent back to the app in the field. Such platform will require a digitalization system with demonstrated optical resolution and robustness to be deployed in the field, mobile connectivity and enough bandwidth to upload the compressed images, and enough on-line examiners to provide a fast analysis. Although the proposed system needs some level of expertise to prepare and acquire the images, once the slide is prepared, the process of image digitalization and transmission over the mobile network can be done with a specific app that is easy to use and does not require specific training for the user. Image regions with red blood cells can be cropped manually after the digitalization process in the same app or could be automatically detected by a machine learning algorithm. In a field test done by our team in Mozambique, the complete process of digitalizing a sample (in this case thick smears), uploading them to the internet, volunteers making the on-line analysis and sending back the results to the field took 15 min [30].

A complete digital diagnosis protocol could be divided in two steps, each one corresponding to a different game. First determine parasitaemia in a thick smear and then perform the species differentiation and also evaluate the possibility of a mixed infection.

These results are in line with previous works that required other visual tasks over medical images. As a by-product of our experimental setting, it was measured the time that takes to decide which is the species, which is in the order of 2–3 s. Such a short time reinforces the idea that it is possible and easy to train non-experts in specific tasks around visual interpretation of biomedical images.

Other approaches, as automatic image processing pipelines [8, 34] and more recently artificial intelligence systems based on neural network models [9, 35] have provided high levels of sensitivity and specificity in malaria image analysis which could allow to detect sub-clinic malaria in microscopic samples. However, one of the main challenges for these type of methods is the need for a massive database with tagged images to train the algorithms for each acquisition environment. A hybrid system that combines on-line volunteers tagging images in a particular acquisition environment and artificial intelligence models that adapt based on collaborative decisions will increase the performance of any of the existing approaches individually.

## Conclusions

In conclusion, this work demonstrates that non-expert gamers can easily and rapidly learn the process of malaria classification, producing an accurate collective species classification when results from different players are aggregated. While more work needs to be done to deploy an operational solution, this method could offer a universally available solution to achieve a rapid diagnosis of the malaria species [30] for those areas with mobile connectivity and without microscopy expertise.

## Additional files

**Additional file 1.** (a–e) Tutorial images used before beginning each level showing the characteristic shape and size of each malaria specie in the different levels of the game: a) *P. falciparum,* b) *P. vivax*, c) *P. ovale*, d) *P. malariae*, e) *P. knowlesi*. (f–g) Feedback given to the players after every success (f) or mistake (g), showing the right solution. (h) Review of the score obtained and the number of puzzles achieved at the end of a level.

**Additional file 2.** (a) Percentage of hits scored in the different intervals of opened windows for the different gamers. (b) Time to answer as gamers opened new game windows. (c) Percentage of true (green) or false (red) answers when gamers have beaten the four levels of difficulty (arcade +) or not (arcade−). Values given represents the mean ± SEM. ***P ≤ 0.0001.

## Abbreviations

ACT: artemisinin-based combination treatment
ANOVA: analysis of variance
HRP2: histidine-rich protein 2
PCR: polymerase chain reaction
