# Supplementary figures and images for: Collaborative intelligence and gamification for on-line malaria species differentiation
Source: Malar J. 2019 Jan 24;18:21. doi: 10.1186/s12936-019-2662-9 (PMC6345056; doi:10.1186/s12936-019-2662-9)

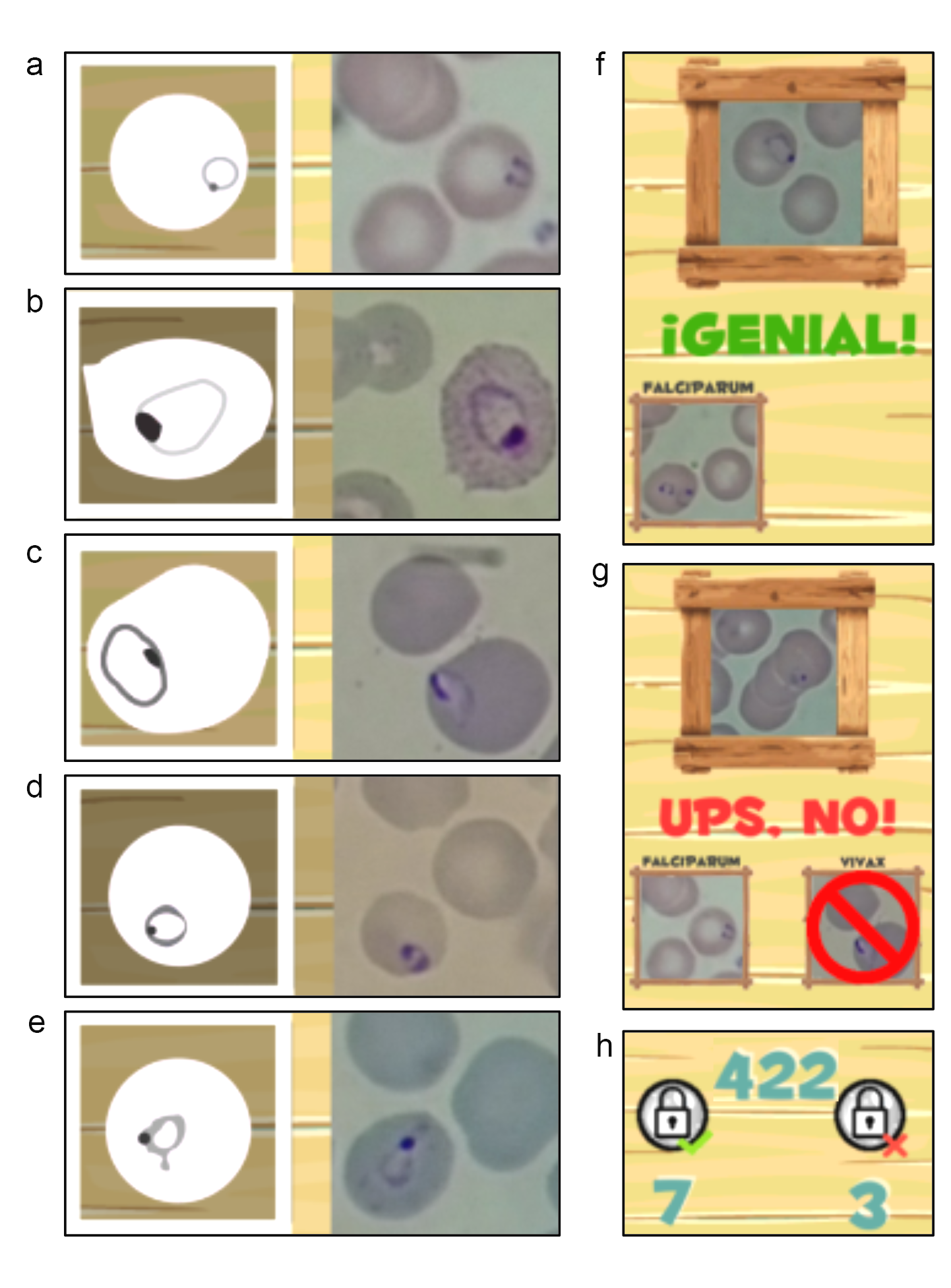

Supplement: Supplementary file 1 — Additional file 1. (a–e) Tutorial images used before beginning each level showing the characteristic shape and size of each malaria specie in the different levels of the game: a) P. falciparum, b) P. vivax, c) P. ovale, d) P. malariae, e) P. knowlesi. (f–g) Feedback given to the players after every success (f) or mistake (g), showing the right solution. (h) Review of the score obtained and the number of puzzles achieved at the end of a level. [file 12936_2019_2662_MOESM1_ESM.tif]

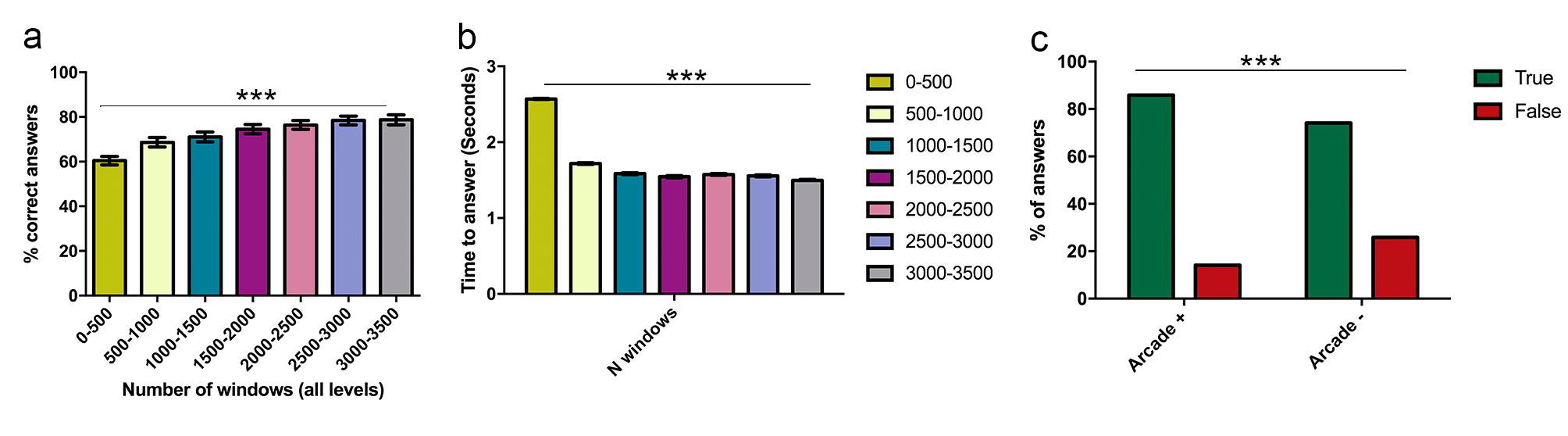

Supplement: Supplementary file 2 — Additional file 2. (a) Percentage of hits scored in the different intervals of opened windows for the different gamers. (b) Time to answer as gamers opened new game windows. (c) Percentage of true (green) or false (red) answers when gamers have beaten the four levels of difficulty (arcade +) or not (arcade−). Values given represents the mean ± SEM. ***P ≤ 0.0001. [file 12936_2019_2662_MOESM2_ESM.tif]
